# Guilt as a Motivator for Moral Judgment: An Autobiographical Memory Study

**DOI:** 10.3389/fpsyg.2017.00750

**Published:** 2017-05-10

**Authors:** Igor Knez, Ola Nordhall

**Affiliations:** Department of Social Work and Psychology, University of GävleGävle, Sweden

**Keywords:** guilt, moral intention, the self, autobiographical memory

## Abstract

The aim was to investigate the phenomenology of self-defining moral memory and its relations to self-conscious feelings of guilt and willingness to do wrong (moral intention) in social and economic moral situations. We found that people use guilt as a moral motivator for their moral intention. The *reparative* function of guilt varied, however, with type of situation; that is, participants felt guiltier and were less willing to do wrong in economic compared to social moral situations. The self-defining moral memory was shown to be relatively *more* easy to access (accessibility), logically structured (coherence), vivid, seen from the first-person perspective (visual perspective), real (sensory detail); but was relatively *less* positive (valence), emotionally intense, chronologically clear (time perspective), in agreement with the present self (distancing), and shared. Finally, it was indicated that the more guilt people felt the more hidden/denied (less accessible), but more real (more sensory details), the self-defining moral memory.

## Introduction

“I think, therefore I am.” With these words Descartes illustrates a fundamental duality of the self. That is, a polarity of two metaphysically separate entities (the *knower*, “I” – ontological perspective; and the *known*, “me” – epistemological perspective) that interact in emitting the phenomenological experience of being someone; a person including a self (Kant, 1787/1929; James, 1890/1950; Metzinger, 2009; Klein, 2014). In the words of Kant (1787/1929, p. 35): “… through inner *experience* I am conscious of *my existence* in time, and this is more than to be conscious merely of my representation. It is identical with the *empirical consciousness of my existence ….*”

Thus, representing ourselves mentally suggests that we are *what* we remember (Kihlstrom and Klein, 1994; Wilson and Ross, 2003; Conway and Holmes, 2004; Singer, 2005). In view of this, Locke (1690/1849) assumed that *what* we do *not* remember is *not* part of ourselves. Quite the reverse, Hume (1739/1967) stated that memory does not *re-play* in any precise manner but *re-constructs* our stored personal experiences; a view that is in line with present cognitive psychology (e.g., Schacter and Addis, 2007).

The function of a personal, autobiographical, memory is to ground the self and its social position as well as to regulate the self’s current and future behaviors, goals and problem solving (Neisser, 1988; Brewer, 1999; Habermas and Bluck, 2000; Pillemer, 2003; Conway, 2005). According to Fivush et al. (2011, p. 323): “… autobiographical memory is at the heart of human understanding of self and other, as the way in which individuals create a sense of self as continuous and coherent through time, with a past that explains the present and projects into the future….” This comprises conceptual and personal types of information and comprehension about oneself and its identity, apportioned across declarative memory; including self-related schemas, scripts, values, attitudes, goals, and beliefs as well as personal reminiscence and sensory information shared socially as life narratives (Kihlstrom et al., 2003; Conway et al., 2004; Klein et al., 2004; Thomsen, 2009; Klein, 2012; Knez, 2014, 2016a; Knez and Eliasson, 2017; Knez et al., 2017).

In addition, phenomenology is a crucial feature of our life narratives because autobiographical memories can generate strong phenomenological experiences, thoughts, and emotions (Singer and Salovey, 1993; Sutin and Robins, 2007). Phenomenology provides subjective sense of the self too (Prebble et al., 2013) by motivating the self to mentally travel through the mind; in the words of Tulving (2005, p. 15): “…there can be no travel without a traveler.” This results in a feeling of a lifelong existence through time (Kant, 1787/1929; Klein et al., 2004; Addis and Tippett, 2008). Several dimensions of phenomenology have also been shown, for example, to regulate emotions (Raes et al., 2003), influence self-change (Libby et al., 2005) and goal attainment (Singer and Salovey, 1993).

### Morality of the Self

As shown above the self is linked to memory (Kihlstrom and Klein, 1994; Wilson and Ross, 2003; Conway, 2005; Klein, 2012), but also to its morality via self-conscious emotions of, for example, guilt. A self-conscious, compared to, for example, a basic, emotion is related to the self because it can be elicited only by the self: “… self-awareness… can affect the nature of emotional experience… it can bring self-evaluative states such as shame, guilt, and pride” (Silvia and Eddington, 2012, p. 426). Guilt is, in other words, elaborated by the cognitions of the self; by the “I” and “me” self-reflections and evaluations (Tracy and Robins, 2004), in relation to some personal and/or cultural standard (Lewis, 2008). Its adaptive, *reparative*, function is to motivate the psychological agent to avoid socially undesirable behavior (Baumeister et al., 1994; Cristofari and Guitton, 2014); that is, *not* “to do wrong”.

In that sense, guilt is part of the *morality* of the self, the autobiographical memory (Körner et al., 2016), due to its psychological role of blaming one’s behavior: “I *did* that horrible *thing*” (Tangney and Tracy, 2012, p. 448). Several previous findings in emotion research have also indicated that one of the roles of negative emotions is to regulate behavior (Frijda et al., 1989; Scherer and Wallbott, 1994). It has also been suggested that *proneness* to guilt may be a trait that prevent people from thinking, feeling and behaving unethically (Cohen et al., 2011, 2012). In other words, “guilt proneness is a personal trait indicative of a predisposition to experience negative feelings about personal wrongdoing” (Cohen et al., 2012, p. 355). It has been shown to differ, for example, between people who have not been diagnosed with antisocial personality disorder and those with antisocial personality disorder (Haidt and Kesebir, 2010). Finally, previous neuroimaging studies have also indicated neural correlates between moral functioning and autobiographical memory (Han, 2017) as well as between moral emotions and self-related processing (Han et al., 2016); thus, suggesting associations between the self and its morality at the neural level.

As mentioned above, remembering is a reconstructive process. This means that recalled personal, autobiographical, memory can transitorily reconstruct one’s sense of the self. For example, Gino and Desai (2012) showed that asking participants to recall childhood memories triggered the concept of “moral purity” (“Children are innocent and virtuous.”); which in turn affected their prosocial behavior. This suggests that our sense of the self may be influenced and momentarily re-structured by moral involvements (Bergman, 2002; Hardy and Carlo, 2005; Heiphetz et al., 2016), indicating a moral self-regulating mechanism (Escobedo and Adolphs, 2010); an essential moral self, suggesting that “Moral traits are considered more important to personal identity than any other part of the mind” (Strohminger and Nichols, 2014, p. 168).

It can, according to Blasi (1984), be experienced phenomenologically as the “real me”: “…the authentic self, and the deepest principle that guides the individual” (Stets and Carter, 2011, p. 194). In line with this, some studies have indicated that recalling good past moral actions can motivate a consistency in subsequent moral behavior (Reed et al., 2007; Shao et al., 2008), but others have shown that good past moral actions can also *license* to act immorally (Monin and Miller, 2001; Sachdeva et al., 2009; Merritt et al., 2010; Conway and Peetz, 2012).

### Present Study

Given the above, we investigated one aspect of the reconstructive personal, autobiographical memory, namely, the past *self-defining moral memories* in relation to present moral intentions of *willingness to do wrong* and feelings of *guilt* in social and economic situations. We posed the following questions: how do we remember our own moral deeds, and do they have any impact on our current morality in similar contexts? In general, we expected the self-defining moral memory to be coherent, vivid and easy to recall due to its first-person perspective and the self-conscious, emotional constituents (Moll et al., 2005; Welzer and Markowitsch, 2005; Escobedo and Adolphs, 2010; Sutin and Robins, 2010), as well as involving the feeling of “seamless whole” (Prebble et al., 2013). In line with we-are-motivated-to-act-consistent-with-our self-view perspective (Blasi, 1980; Aquino and Reed, 2002), we also predicted that the moral intention of willingness to do wrong will be lower in participants when they experienced greater feelings of guilt, due to the latter’s reparative function (Tangney et al., 1996; Gino and Pierce, 2009).

The two types of situations were included because previous research has indicated social (Kortenkamp and Moore, 2006; Greenwood, 2011; Knez, 2013, 2016b) and economic/resource (Hardin, 1968; Agerström and Björklund, 2009) dimensions of moral dilemmas; in the words of Aquino et al. (2009, p. 124): “…situational factors may activate a person’s moral identity or they may activate alternative facets of identity, thereby increasing or decreasing the current accessibility of the moral self-schema within the working self-concept.” Some previous research has also indicated that morality and self-conscious emotions may differ across cultures (Markus and Kitayama, 1991; Wang, 2008; Wong, 2009). For example, Vauclair et al. (2014) investigating four-country/two-culture samples reported some type of relativism in morality dimensions across cultures.

Finally, we measured 10 aspects of self-defining moral memory (vividness, coherence, accessibility, time perspective, sensory detail, emotional intensity, visual perspective, sharing, distancing, and valence) previously identified as the phenomenological dimensions of autobiographical memory (Sutin and Robins, 2007). Phenomenology is a crucial feature of autobiographical memory providing experiences, thoughts and emotions (Singer and Salovey, 1993; Sutin and Robins, 2007) as the base for the sense of self (Prebble et al., 2013).

## Materials and Methods

### Participants

In total, 116 subjects with varying levels of education and life experiences and a mean age of 25.9 (range = 17–67) participated in the present study. They (90 women and 26 men) were randomly recruited from a university (*N* = 63 students, females = 51, males = 12, mean age = 23.7, age range = 18–38), an upper secondary school (*N* = 32 students, females = 28, males = 4, mean age = 18.2, age range = 17–20), and from a private company (*N* = 21 employees, females = 11, males = 10, mean age = 44.4, age range = 26–67). The subjects received a cinema ticket in appreciation of their participation.

### Procedure

The temporal ordering of the tasks began with the judgment and guilt estimations of moral dilemmas followed by the estimations of subjects’ most important self-defining moral memories; memories of high “moral identity centrality” (Aquino et al., 2009; Dinh and Lord, 2013) grounding the self (Conway, 2005).

First, participants were told that their participation was voluntary and that they could leave the study whenever they wanted. Second, participants completed the moral judgment task (willingness to do wrong and anticipated guilt) including four dilemmas, two representing a social and two representing an economic situation (see below for details). Third, they were asked to recall and write down their most important self-defining moral memory (by using one the four dilemmas as a frame of reference for their self-defining moral memory; this was done, in order to relate estimations of moral intention and guilt to self-defining moral memory) with the following instruction:

“Think for a couple of minutes about your most important moral memory that resembles one of the dilemmas depicted in the moral judgment task 1–4. Describe it in detail by writing down what you remember: what happened and when, who you were with (if anyone), and how you felt and reacted. It may be a memory about any kind of experience, but it should be a memory of moral importance that you have thought about many times and that is still important to you, even as you recall it now. This memory should also be central in understanding who you are, your identity”.

Fourth, they were asked to estimate their retrieved self-defining moral memory (see above) on 63 scales, measuring the phenomenological dimensions of vividness, coherence, accessibility, time perspective, sensory detail, emotional intensity, visual perspective, sharing, distancing, and valence. In the present study we will report only quantitative data; that is, the phenomenology estimations (63 scales) of the self-defining moral memory.

Finally, an ethical approval was not required for this study in accordance with national and institutional guidelines. Though, we conducted it in accordance with APA’s (American Psychological Association) ethics code. Accordingly, participants were informed about: (1) objectives of the present study; (2) their right to withdraw from the participation at any time without any consequences; (3) how long it will take to complete the tasks and information about the types of tasks involved; (4) confidentiality; (5) that they will not be financially compensated for their participation; and (6) whom to contact about any questions related to this study.

### Measures

In line with Agerström and Björklund (2009), behavioral moral intentions *(willingness to do wrong)* and anticipated moral guilt *(feelings of guilt)* were measured with four moral dilemmas; two (nr. one and three, see below) depicting *social*, and two (nr. two and four, see below) depicting *economic* situations. Moral dilemmas represented a disagreement between altruistic and egoistic motives (Agerström and Björklund, 2009; Knez, 2016b) in that the participants faced a conflict between unselfish motives in terms of greater well-being or gain for one or several other persons (at the cost of a smaller personal discomfort or financial lost) and selfish (pure hedonic) motives in terms of a smaller personal comfort, convenience, or gain (at the cost of a greater utility, well-being or pro-environmental values for one or several other persons). Thus, dilemmas depicted a clash between universal and non-universal norms/principles/values (Hare, 1981; Hauser, 2006). Accordingly and yielding all dilemmas, participant faced a conflict between two alternatives; one implying worse consequences for one or several other people, and one implying better outcomes compared to the alternatives (Singer, 2011).

Participants were asked to indicate their willingness to do wrong and feelings of guilt on a nine-point Likert scale, where 1 indicated “not likely at all” and “no guilt at all” and 9 indicated “very likely” and “extreme guilt.” The following four dilemmas and subsequent two questions, measuring moral intention and moral guilt respectively, were administrated:

(1) You are about to throw your garbage. The weather is cold and rainy and there is no roof covering the trash cans. You have not sorted the garbage and now you realize that it will get you standing out in the rain sorting the garbage in order to get it in the cans.
Moral *intention* question: how likely do you think it is that you will throw the garbage without recycling it?
Moral *guilt* question: to what extent would you feel guilty if you do not recycle your garbage?
(2) You find a bag containing 800 SEK (approximately 80 Euros) together with information about the owner. You consider all nice things that you could buy if you keep the money instead of contacting the owner.
Moral *intention* question: how likely do you think it is that you will keep the money?
Moral *guilt* question: to what extent would you feel guilty if you did not hand back the money?
(3) It is Saturday and the first day for a long time since you were off duty. A colleague who you usually associate with needs help moving to a new apartment and asks you for assistance. Your colleague has just received a cancelation from another person. You realize that the move will be laborious and tedious. At the same time you know that some of your friends are about to spend Saturday outdoors, enjoying nice weather and tasty food.
Moral *intention* question: how likely do you think it is that you will hang out with friends instead of helping your colleague to move?
Moral *guilt* question: to what extent would you feel guilty if you did not help your colleague to move?
(4) You are just about to do your income-tax return. Given that you work in another town you have the legal right to do tax discounts for the trips to the work. You realize that it easy for you to discount for more than you have the legal right to. The only thing you need to do is to declare that you have used your own car although you and your colleague have been taking turns to drive.
Moral *intention* question: how likely do you think it is that you would have made a larger deduction than you are entitled to do?
Moral *guilt* question: to what extent would you feel guilty if you had made a larger deduction than you are entitled to do?

“The Memory Experience Questionnaire” (Sutin and Robins, 2007), henceforth MEQ, was used to estimate subjects’ most important self-defining moral memories. This measure contains 63 scales/statements, defining the 10 phenomenological dimensions of vividness, coherence, accessibility, time perspective, sensory detail, emotional intensity, visual perspective, sharing, distancing, and valence. Estimations were made on a five-point Likert scale ranging from 1 “strongly disagree” to 5 “strongly agree.” The recalled self-defining moral memories were categorized as belonging to either a “self-defining moral memory related to a *social* situation (e.g., betrayal of a desperate friend)” or a “self-defining memory related to an *economic* situation (e.g., tax evasion).”

The 10 phenomenological dimensions of MEQ have previously shown an adequate reliability, Cronbach alphas of 0.72 to 0.97 (Sutin and Robins, 2007). According to these authors, *vividness* (Cronbach alpha 0.85) refers to the visual clarity and intensity of a memory. *Coherence* (Cronbach alpha 0.79) implies the degree to which the memory is remembered as a logical story in a certain time and place rather than fragments of an experience or a mixture of similar memories. *Accessibility* (Cronbach alpha 0.83) indicates the easy of a memory to be retrieved. *Time perspective* (Cronbach alpha 0.85) refers to the perceived time-related clarity. *Sensory detail* (Cronbach alpha 0.72) measures the sensory details re-experienced. *Emotional intensity* (Cronbach alpha 0.86) refers to the intensity of memory-related emotions. *Visual perspective* (Cronbach alpha 0.87) indicates the perspective from which the memory is “seen”; higher scores indicate that the person views the experience through her/his own eyes (a first person perspective) as opposed to being an observer watching oneself (a third person perspective). *Sharing* (Cronbach alpha 0.89) measures the extent to which a memory is shared with others. *Distancing* (Cronbach alpha 0.87) measures how much the person distances him-/herself from the retrieved memory, that is, from “having been” that person in the memory. *Valence* (Cronbach alpha 0.97) indicates the positive/negative valance of a memory.

### Design and Data Analyses

The within-subject-independent variables were: (1) moral-related intention in social vs. economic situations; (2) moral-related guilt in social vs. economic situations; and (3) 10 phenomenological dimensions of the self-defining moral memory. The between-subject-independent variable was the self-defining moral memories categorized as belonging to either a “self-defining moral memory related to a *social* situation (e.g., betrayal of a desperate friend)” or a “self-defining memory related to an *economic* situation (e.g., tax evasion).

The dependent variables were the estimations of willingness to do wrong (moral-related intention), feelings of guilt (moral-related guilt), and phenomenology of the self-defining memory.

Five types of analyses was performed: (1) Effects of self-defining moral memory (memories of social vs. economic moral situations) and moral-related intention (willingness to do wrong in social vs. economic moral situations) on moral-intention-estimation; (2) Effects of self-defining moral memory (memories of social vs. economic moral situations) and moral-related guilt (feelings of guilt in social vs. economic moral situations) on moral-guilt-estimation; (3) Associations between feelings of guilt and moral intention (willingness to do wrong) in social vs. economic moral situations; (4) Effects of self-defining moral memory (memories of social vs. economic moral situations) and 10 phenomenological dimensions on mean phenomenology of self-defining memory; and (5) Associations between moral-related intention (willingness to do wrong) and guilt, and the 10 phenomenological dimensions of self-defining moral memory, across moral situations. ANOVAs were performed in sections (1), (2), and (4). Regression analyses were performed in sections (3) and (5). Note: no significant correlations between age and estimations of moral intention and guilt were found; therefore the age was excluded from further analyses.

Additionally, and following Sutin and Robins (2007) a descriptive interpretation of the mean phenomenology (estimation) for *each* dimension was made. Thus, and in addition to the parametric statistics, we interpreted the mean estimation *within* each dimension as signifying *more* (mean score 3-5) or *less* (mean score 3-1) type of phenomenology (10 dimensions). For example, a self-defining moral memory was interpreted as *more* easy to access (accessibility) and share with others (sharing) *if* the mean estimation was 3-5 and as *less* easy to access and share with others *if* the mean estimation was 3-1. In other words, the mean score of 3 on a five-point Likert scale was regarded as a demarcation line of a *more* or *less* type of a phenomenology of self-defining moral memory content.

## Results

In line with the five types of data analyses, suggested in Section “Design and Data Analyses,” the results will be reported in five sections related to these analyses.

### Self-Defining Moral Memory and Moral-Related Intention (Willingness To Do Wrong)

No significant result involving self-defining memory was obtained. However, a main effect of type of moral-related intention (willingness to do wrong), Greenhouse–Geisser = 2.98 (*MSE*), *F*(1,114) = 74.57, *p* < 0.01, η^2^ = 0.4, showed that participants were more willing to do wrong in social compared to economic moral situations (*M* = 5.47, *SD* = 1.84 vs. *M* = 3.51, *SD* = 1.91).

### Self-Defining Moral Memory and Moral-Related Guilt (Feelings of Guilt)

As above, no significant result involving self-defining memory was obtained. A main effect of type of moral-related guilt, Greenhouse–Geisser = 2.14 (*MSE*), *F*(1,114) = 26.25, *p* < 0.01, η^2^ = 0.19, showed however, that participants felt more guilty when behaving immorally in economic vs. social moral situations (*M* = 6.43, *SD* = 1.76 vs. *M* = 5.45, *SD* = 1.72).

### Relation between Feelings of Guilt and Moral Intention (Willingness To Do Wrong) in Social vs. Economic Moral Situation

Given the two results above, regression analyses were performed to check for the associations between guilt and moral intention (willingness to do wrong) in the respective moral type of situation. As can be seen in **Table 1**, guilt was a stronger predictor for willingness to do wrong in economic compared to social moral situations. Thus, the association *the higher the level of guilt the less willingness to do wrong* was accounted for more by the economic than by the social moral situation (explained variance 50% vs. 24%, see **Table 1**).

**Table 1:** Regression statistics for the relation between feelings of guilt as predictor and moral intention (willingness to do wrong) as criterion variable in *social* and *economic* moral situations respectively.

*R*^2^	Beta(β)	*SE*	df	MS	*F*	*t*	Significance
0.24 (social)	-0.49	0.08	1, 115	80.42	35.30	-5.94	0.00
0.50 (economic)	-0.70	0.06	1, 115	176.18	112.04	-10.59	0.00

### Self-Defining Moral Memory and Its Phenomenology

**FIGURE 1:**
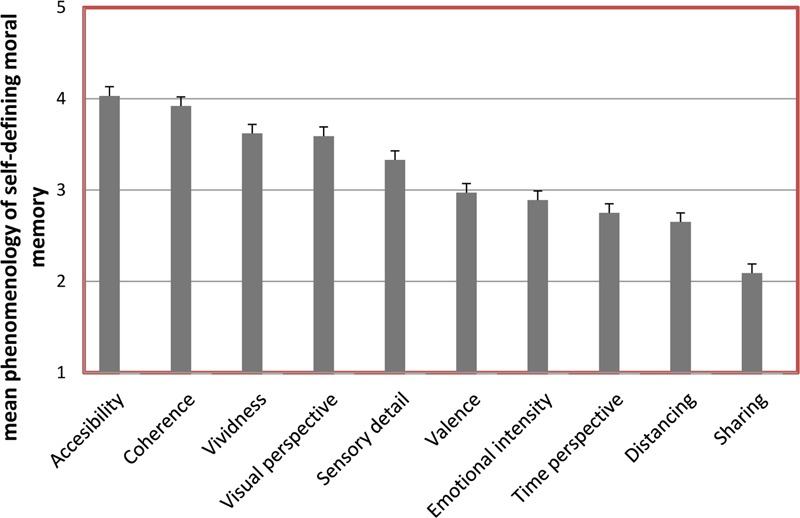
**Mean phenomenology estimations in self-defining moral memory, comprising dimensions of accessibility, coherence, vividness, visual perspective, sensory detail, valence, emotional intensity, time perspective, distancing, sharing.** Error bars represent SE.

A main effect of the 10 phenomenological dimensions, Greenhouse–Geisser = 7.75 *(MSE*), *F*(9,1026) = 143.48, *p* < 0.01, η^2^ = 0.14, showed that participants’ self-defining moral memory contained mostly details of accessibility (*M* = 4.05, *SD* = 3.67) and least of sharing (*M* = 2.08, *SD* = 0.93), *t*(115) = 5.59, *p* < 0.01 (see **Figure 1**). This indicates that the self-defining moral memory is generally easy to retrieve, but rarely shared with others (*p* < 0.01). (For all *post hoc* comparisons between the 10 phenomenological dimensions see **Table 2**.)

**Table 2:** *p*-values for the pairwise *post hoc* comparisons (LSD) between the 10 phenomenological dimensions of self-defining moral memory.

Phenomenological dimensions of self-defining moral memories	A	C	V	VP	SD	VA	EI	TP	D	SH
Accessibility (A)		0.73	0.20	0.18	0.04	0.00	0.00	0.00	0.00	0.00
Coherence (C)	0.73		0.00	0.01	0.00	0.00	0.00	0.00	0.00	0.00
Vividness (V)	0.20	0.00		0.79	0.00	0.00	0.00	0.00	0.00	0.00
Visual perspective (VP)	0.18	0.01	0.79		0.02	0.00	0.00	0.00	0.00	0.00
Sensory detail (SD)	0.04	0.00	0.00	0.02		0.01	0.00	0.00	0.00	0.00
Valence (VA)	0.00	0.00	0.00	0.00	0.01		0.59	0.11	0.16	0.00
Emotional intensity (EI)	0.00	0.00	0.00	0.00	0.00	0.59		0.37	0.30	0.00
Time perspective (TP)	0.00	0.00	0.00	0.00	0.00	0.11	0.37		0.68	0.00
Distancing (D)	0.00	0.00	0.00	0.00	0.00	0.16	0.30	0.68		0.01
Sharing (SH)	0.00	0.00	0.00	0.00	0.00	0.00	0.00	0.00	0.01	

However, if we interpret descriptively (see Materials and Methods) the mean phenomenology *within* each dimension (signifying 3-5 as *more* and 3-1 as *less*), it can tentatively be suggested that (see **Figure 1**) the self-defining moral memory is relatively: (1) *more* easy to access (accessibility), logically structured (coherence), vivid, seen from the first-person perspective (visual perspective), real (sensory detail), and (2) *less* positive (valence), emotionally intense, chronologically clear (time perspective), in agreement with the present self (distancing), told about to others (sharing).

**FIGURE 2:**
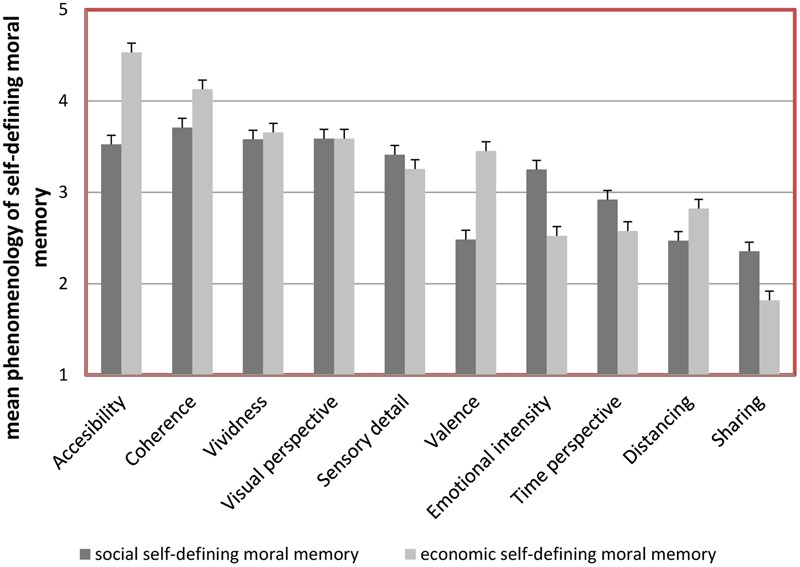
**Mean phenomenology estimations (comprising dimensions of accessibility, coherence, vividness, visual perspective, sensory detail, valence, emotional intensity, time perspective, distancing, sharing) in social vs. economic self-defining moral memory.** Error bars represent SE.

Additionally, an interaction effect between the type of self-defining moral memory and the 10 phenomenological dimensions, Greenhouse–Geisser = 143.87 (MS), *F*(9,1026) = 18.67, *p* < 0.01, η^2^ = 0.14, was obtained. According to the follow-up one-way ANOVAs significant differences between social and economic category of self-defining moral memory were shown for valance, *F*(1,116) = 19.22, *p* < 0.01, η^2^ = 0.14, emotional intensity, *F*(1,116) = 10.02, *p* < 0.01, η^2^ = 0.08, sharing, *F*(1,116) = 10.26, *p* < 0.01, η^2^ = 0.08, and coherence, *F*(1,116) = 8.95, *p* < 0.01, η^2^ = 0.07. As can be seen in **Figure 2**, this indicates that the self-defining social compared to economic moral memory is *more* negative, emotionally intense and talked about, but *less* coherent.

### Phenomenology of Self-Defining Moral Memory and Moral-Related Intention (Willingness To Do Wrong) and Guilt

Regression analyses were performed to check for the associations between moral-related intention (willingness to do wrong) and guilt, across moral situations, and the 10 phenomenological dimensions of self-defining moral memory. It was shown that the higher willingness to do wrong the *less* the accessibility of self-defining moral memory, and the greater the feeling of guilt the *more* real (sensory detail) self-defining moral memory (see **Table 3**).

**Table 3:** Regressions statistics for the relation between moral intention (willingness to do wrong across moral situations) as predictor and *accessibility* (A) and *sensory detail* (SD) respectively of the self-defining moral memory as criterion variable.

*R*^2^	Beta(β)	*SE*	df	MS	*F*	*t*	Significance
0.03 (A)	-0.18	0.04	1, 115	7.94	3.99	-2.0	0.00
0.04 (SD)	0.19	0.15	1, 115	8.15	4.27	2.07	0.00

## Discussion

In line with our prediction, we found that people use the self-conscious feeling of guilt as a moral motivator for their moral intention of willingness to do wrong (Tangney et al., 1996; Tracy and Robins, 2004; Lewis, 2008; Gino and Pierce, 2009; Tangney and Tracy, 2012). The reparative function of guilt varied, however, with the type of situation. That is to say, participants felt guiltier and were less willing to do wrong in economic compared to social moral situations. Accordingly, the association *the higher the level of guilt the less willingness to do wrong* was accounted for more by the economic than the social moral situation, with an explained variance of 50% vs. 24% (see **Table 1**).

Given that self-conscious emotions such as guilt are “evoked in situations where a person’s behavior or traits are deemed discrepant from social or moral standards” (Lickel et al., 2014, p. 1050) and that the self, morality and self-conscious emotions differ across cultures (Markus and Kitayama, 1991; Wang, 2008; Wong, 2009; Vauclair et al., 2014), due to the culture’s “directive” (the “good” person standards) and “evocative” (emotions that are “allowed” to be expressed/experienced in a specific situation) functions (Cross and Gore, 2012), we may tentatively interpret these results as yielding a cultural effect. In other words, the north European Protestant culture (Swedish participants) seems to promote higher feelings of guilt and by that less moral intention of willing to do wrong in economic compared to social moral situations; indicating the latter type of situations as less socially regulated and the role of guilt, as negative emotion, to regulate behavior (Frijda et al., 1989; Scherer and Wallbott, 1994). Generally, this is also in line with some previous research indicating social (Kortenkamp and Moore, 2006; Greenwood, 2011; Knez, 2013, 2016b) and economic/resource (Hardin, 1968; Agerström and Björklund, 2009) dimensions of moral dilemmas; situational aspects that may activate different facets of the moral self (Aquino et al., 2009).

As predicted, the self-defining moral memory was shown to be easy to access (Welzer and Markowitsch, 2005; Escobedo and Adolphs, 2010), but was less shared with others. Several previous studies have shown that self-conscious (moral) emotion of shame compared to guilt may lead to denial and hiding; whereas guilt may trigger processes of confessing and amending (Tangney and Tracy, 2012). Our data indicate, however, that self-defining *moral* memory *per se* may be less shared with others (“hided, denied”), which is in line with previous shame-related findings (Tangney et al., 1996).

The *within*-phenomenological-dimension-interpretation of the self-defining moral memory revealed (see **Figure 1**), furthermore, that this kind of memory was relatively *more* easy to access (accessibility), logically structured (coherence), vivid, seen from the first-person perspective (visual perspective), real (sensory detail), relatively *less* positive (valence), emotionally intense, chronologically clear (time perspective), in agreement with the present self (distancing), talked about with others (sharing). This indicates high “moral identity centrality,” meaning that these memories are well-articulated within the overall concept of the self (Conway, 2005; Aquino et al., 2009), and that the dimensions of self-coherence and self-continuity might be important in promoting morality in the self-system (Hershfield et al., 2012). They are fundamental for our feeling of being a “seamless” entity. Accordingly, these processes “unify disparate experiences, levels of consciousness, behaviors, cognitions, and mental representations into a coherent, unified whole” (Prebble et al., 2013, p. 818). Furthermore, self-defining moral memories were shown to be more vivid. All this is in line with previous research showing that autobiographical memories seen from the first-person perspective (vividness) are more coherent, accessible and real (Sutin and Robins, 2010).

However, our results showed that several phenomenological dimensions in self-defining moral memory varied across the two types of situational memory (see **Figure 2**). Given that participants were less willing to do wrong in economic compared to social moral situations; probably, for that reason their economic vs. social self-defining moral memories were shown to be more positive, coherent, and less emotionally intense. If we assume that different situations might operate as different moral prototypes (Walker and Hennig, 2004) including specific behavioral information, then these results are in accordance with, for example, Osswald et al. (2010, p. 1078) findings suggesting that we “associate different moral behaviors with different moral prototypes and that certain moral behavior can be activated by the priming of the related prototype”.

Finally, a link between the moral intention of willingness to do wrong and the self-defining moral memory was indicated, showing that the *more* we do wrong the *less* accessible is our self-defining moral memory, and the *more* we feel guilty the *more* real is our self-defining moral memory comprehended (see **Table 2**). Thus, the more guilt the self infers (“I *did* that horrible *thing*.” – Tangney and Tracy, 2012, p. 448) the more it will experience the self-defining moral memory as real (comprising more sensory details). However, the self will at the same time hide/deny it more (it will be less accessible). In other words, the self will hide/deny the moral memory from itself and others by extrapolating its morality from a bad *thing* to a bad *self* – from “I *did* that horrible *thing*.” to “*I* did that horrible thing” (Tangney and Tracy, 2012, p. 448). All this implies relationships between the self and its morality, as it is correspondingly indicated at the neural level (Han et al., 2016; Han, 2017).

## Author Contributions

Most contribution is made by the first author, IK. The second author, ON, collected data and wrote most parts of the Section “Materials and Methods.”

## Conflict of Interest Statement

The authors declare that the research was conducted in the absence of any commercial or financial relationships that could be construed as a potential conflict of interest.
